# Site-specific labelling of native peptides and proteins: chemical and enzymatic strategies

**DOI:** 10.3762/bjoc.22.67

**Published:** 2026-06-03

**Authors:** Antonio Angelastro, Jonathan Bargh, Subhajit Guria, Victor Laserna, Louis Luk

**Affiliations:** 1 Biologics Engineering, Oncology R&D, Cambridge Biomedical Campus, AstraZeneca, 1 Francis Crick Ave, Trumpington, Cambridge, CB2 0AA, UK; 2 Hit Discovery, Discovery Sciences, R&D, AstraZeneca, Pepparedsleden 1, Mölndal, Gothenburg, 43183, Sweden; 3 Interdisciplinary Research Center, Division of Interdisciplinary Science, Institute for Radiation Sciences, The University of Osaka, 1-2 Machikaneyama-cho, Toyonaka 560-0043, Osaka, Japan; 4 School of Chemistry and Cardiff Catalysis Institute, Cardiff University Main Building, Park Pl, Cardiff, CF10 3AT, UK

**Keywords:** affinity-guided chemistry, disulfide-rebridging, enzyme catalysis, glycan modifications, native-sequence, protein labelling, small-molecule modifications, terminal

## Abstract

Site-specific modifications of native-sequence proteins are technologies that underpin progresses in chemical biology, diagnostics and next-generation biotherapeutics. However, the pursuit of site-specificity has often come at the expense of scalability and usability, ultimately limiting translational potential of a modification tool. This review critically compares current key strategies, including terminal, disulfide-rebridging, small-molecule, glycan and enzyme-based modifications. Finally, we present a decision tree for method selection and highlight opportunities for innovation in next-generation native-sequence protein modification technologies.

## Introduction

Protein and peptide modifications introduce functionalities not achievable through recombinant methods alone, making them vital for fundamental research and biomedical applications [1–4]. Such chemistry underpins wide-ranging biological investigations including microscopy, proteomic analyses and tissue sampling. Their translational power is evident from the success of biotherapeutics: antibody–drug conjugates (ADCs), in which cytotoxic payloads covalently linked to targeting antibodies, are at the forefront of cancer therapy, delivering tumour-selective killing for improved patient outcomes; Neulasta, an *N-*terminal PEGylated granulocyte colony-stimulating factor (G-CSF), is administered to reduce risk of neutropenia in patients receiving chemotherapy. Despite clinical successes, unsolved technical challenges remain. Taking ADC development as an example, a single payload can be insufficient to overcome resistance, highlighting the need for complementary site-specific antibody labelling strategies. Meanwhile, advances in high-throughput screening and AI-guided design have been creating new scaffolds, including nanobodies, de novo binders and peptoids, where tailored modification chemistries have strong potential to generate novel biotherapeutics.

As the development of protein-based therapeutics and diagnostics continues to expand to address global health challenges, the demand for tailored protein-modification strategies is increasing in parallel. Indeed, protein-modification chemistry is a key area of chemical biology in which academic discoveries can rapidly advance from fundamental research (technology readiness level, TRL 1) to patentable, preclinical-stage technologies (TRL 4–5), with some ultimately reaching real-world applications approaching TRL 9. The ADC market alone is projected to reach USD 32 billion by 2034 (https://www.precedenceresearch.com/antibody-drug-conjugates-market), with advanced modalities such as bispecific antibody–drug conjugates (BsADC) gaining traction [2]. Leading suppliers, including ThermoFisher Scientific (Molecular Probes/Invitrogen), LI-COR Biosciences, Roche, Jena Bioscience, Merck (Sigma-Aldrich), New England Biolabs, Promega, Agilent and Cytiva (Danaher), offer a plethora of reagents and kits, from fluorescent tags, crosslinkers to enzymatic tools. Meanwhile, companies such as EnzyTag, Shiru, and Revvity offer bespoke conjugation services. Beyond therapeutics, advances in protein modification have the potential to partially replace solid-phase peptide synthesis (SPPS), reducing environmental impact by minimising protecting groups and harmful solvents. Driven by scientific innovation, clinical demand and sustainability goals, the global protein-modification market is expected to reach USD 6.02 billion by 2034 (https://www.towardshealthcare.com/insights/protein-labeling-market-sizing).

## Review

### Key criteria for effective native-sequence modifications

Effective protein modification relies on tools that balance three interlinked factors translating basic research to commercial applications: site-specificity, usability and scalability.

**Site-specificity.** The ideal modification chemistry targets a single, well-defined site without stochastic off-site reactions. Product homogeneity enhances safety by mitigating toxic by-products that require excessive chromatography, while also offering well-defined pharmacokinetic and pharmacodynamic profiles. Homogeneous batches of labelled proteins thus improve therapeutic indices and reproducibility, key determinants of clinical trial outcomes.

**Usability.** Effective strategies act directly on native proteins, avoiding substrate engineering and minimising risks such as aggregation and immunogenicity, and hence site-specific modifications of commonly found surface residues are desired. Tools are also expected to be easy to prepare and apply, yet remain reliably functional in vitro and in vivo settings. The use of harsh reagents, organic solvents or chaotropic agents must be avoided to preserve protein stability, preventing folding heterogeneity and supporting sustainability.

**Scalability.** For therapeutic and industrial applications, modification methods should minimise waste and curtail costs, reflected in low process mass intensity (PMI). Reagents should be near-stoichiometric, purification steps minimal, and catalysts effective in substoichiometric quantities. Compatibility with one-pot system and automation, such as flow chemistry and robotic synthesis, further enhance scalability.

### Existing tools and techniques

While numerous native-sequence protein-modification tools exist with many being site-specific, few can fulfil all the aforementioned criteria simultaneously. Cysteine modification is among the most effective strategies due to the high reactivity of the thiolate, enabling selective chemical transformations [3–4]. However, the limited abundance of free cysteine residues in proteins has driven the development of alternative approaches targeting other chemical motifs. This review summarizes current strategies for site-specific modifications of these alternative sites, followed by a comparative analysis and outlook on future developments. The strengths, limitations and opportunities are also discussed. Given the diversity and breadth of protein-modification chemistry, more detailed reviews on specific topics are referenced within relevant subsections.

#### Protein terminal modifications

The majority of proteins are linear polypeptide chains, possessing a single *N-* and *C-*terminus and hence presenting these regions as attractive targets for site-specific modification. Indeed, over 80% of protein termini are solvent-exposed and chemically accessible [5]. Notably, the *N-*terminal amine and *C-*terminal carboxyl group exhibit subtle biophysical differences from those of internal residues (lysine, aspartate and glutamate), allowing development of tailored chemistries.

**Scheme 1:**
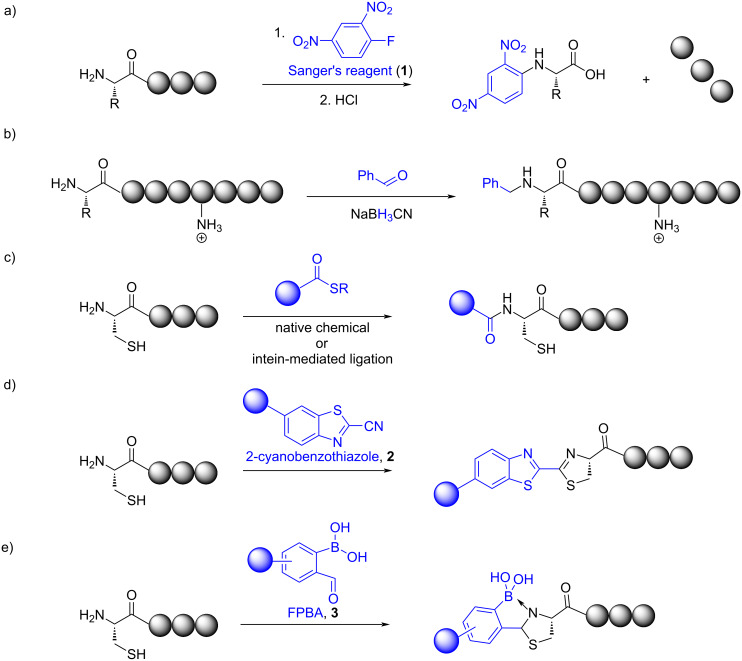
a) Sanger’s reagent (**1**), b) reductive amination by sodium cyanoborohydride, c) native chemical ligation, d) cysteine-reactive 2-cyanobenzothiazole **2** and e) formylphenylboronic acid **3** (FPBA) reacting with *N*-terminal cysteine.

The first chemical modification of an *N-*terminus was reported by Frederick Sanger in 1945 [6], when 1-fluoro-2,4-dinitrobenzene (Sanger's reagent, **1**) was used to label the α-amino group of insulin during sequence determination (Scheme 1a). Its selectivity over lysine’s ε-amine originates from the low p*K*_a_ of the *N-*terminus, influenced by the adjacent carbonyl group and systematically characterized at ≈6–7 (Scheme 1b) [7]. Building on this principle, pH-controlled *N*-terminal PEGylation has been applied to the labelling of filgrastim, producing Neulasta^®^, a long-acting chemotherapy support drug [8].

**Scheme 2:**
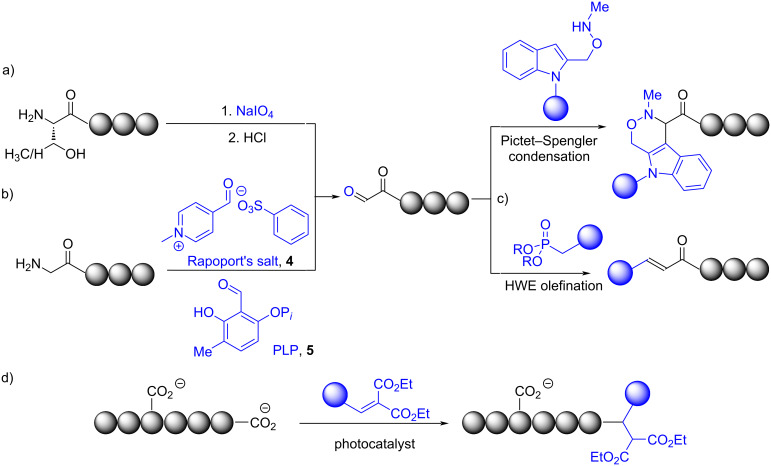
Introduction of carbonyl functionality at protein *N-*termini via a) oxidation by periodate, b) Rapoport’s salt (**4**) or pyridoxal phosphate (PLP, **5**), c) payload attached via Pictet–Spengler or Horner–Wadsworth–Emmons (HWE) reaction, and d) *C-*terminal modification through photoredox catalysis.

Enhanced *N-*terminal modification selectivity can be achieved with dinucleophilic residues. Native chemical ligation recruits *N-*terminal cysteine to form amide bonds via *trans*-thioesterification, though often under denaturing conditions (Scheme 1c) [9]. Intein-mediated ligation is a recombinant alternative but often requires linker sequences. Newer reagents such as 2-cyanobenzothiazole **2** (CBT) react with *N-*terminal cysteine to form luciferin-like linkages (≈9 M^−1^ s^−1^; Scheme 1d) [10], whereas 2-formylphenylboronic acid **3** (FPBA) generates thiazolidino boronate with noticeably improved kinetics (10^3^ M^−1^ s^−1^; Scheme 1e) [11–12]. Aldehydes can be introduced at protein *N-*termini through several strategies: oxidation of serine or threonine with sodium periodate (Scheme 2a) [13], selective modification of glutamate using Rapoport’s salt (**4**) which has generated modified trastuzumab in moderate yield (67%; Scheme 2b) [14], or pyridoxal phosphate (PLP, **5**)-mediated carbonyl formation at residues such as serine, threonine, glycine, alanine, or cysteine [15]. These intermediates enable downstream chemical conjugations such as Pictet–Spengler condensation [16–18] and Horner–Wadsworth–Emmons (HWE) olefination [19] (Scheme 2c).

With respect to *C-*terminal modification, one of the earliest strategies involved the use of intein-mediated protein splicing to generate a *C-*terminal thioester from recombinantly expressed proteins [20–22]. One distinctive feature of the *C-*terminal carboxylate is its lower oxidation potential compared to the side chains of aspartate or glutamate, making it more amenable to selective redox-based modifications. This has led to the development of labelling techniques, wherein the *C-*terminal carboxylate can be functionalized via visible-light photoredox decarboxylative catalysis (Scheme 2d) [23]. Similarly, decarboxylative alkylation has been applied to complex proteomes to enable proteome-wide *C-*terminal profiling [24].

Despite significant advances, major challenges persist. Current *N-*terminal labelling strategies often require engineered residues, operate only under narrow pH conditions, or involve multistep procedures that hinder practical application. Efficiencies often depend on methionine excision, linker or cleavable leader peptides. *C-*Terminal labelling typically suffers from modest conversions (≈50% for photoredox strategies). While selectivity has been improved, few approaches are fully specific. For further discussion, see reviews [25–26].

#### Rebridging agents

**Scheme 3:**
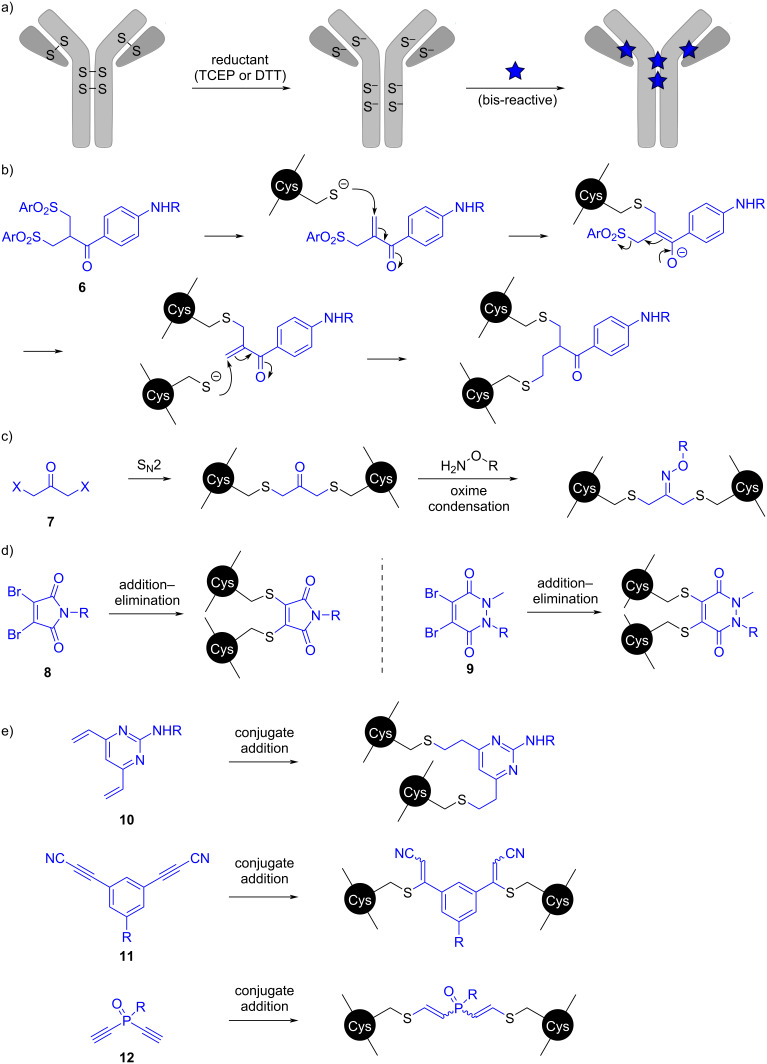
a) Concept of disulfide rebridging. Reagents include b) sulfones **6**, c) dihaloacetones **7**, d) dibromomaleimides **8** and dibromopyridazinediones **9**, e) divinylpyrimidines **10**, arylenedipropiolonitriles **11**, and diethynylphosphonites **12**.

Protein sidechains frequently form complementary pairs, such as disulfide bonds (cystine), salt bridges, hydrogen bonds, and cation–π interactions. Among these, disulfide bonds between cysteine residues are the most widely used for native-sequence protein labelling, due to their unique nucleophilicity and low abundance. Therapeutically relevant proteins, including somatostatin, immunoglobulins (across all isotypes and subclasses), insulin and interleukin-10 naturally contain disulfide bridges. These can be selectively reduced to yield two nucleophilic thiols for reaction with bis-electrophilic reagents to rebridge the cysteines, while introducing a chemical label or crosslinking functionality (Scheme 3a).

One of the earliest rebridging strategies employed bis-sulfone reagents **6** which, upon in situ activation, generate monosulfone Michael acceptors that selectively react with a reduced cysteine pair (Scheme 3b) [27]. This approach was later adapted for native-sequence ADC design on IgG_1_, where four accessible interchain disulfide bonds can be reduced and rebridged to yield conjugates with a drug-to-antibody ratio (DAR) of 4, offering optimal pharmacokinetics and reduced aggregation versus a stochastic maleimide-conjugated antibody. Incorporating cytotoxic payloads into bis-sulfone reagents yielded functional conjugates that exhibited stability in the presence of albumin in vivo [28]. Notably, this technology offers one of the few established routes to generate highly homogeneous ADC candidates, with a moderate level of drug loading, from native antibody sequences.

The bis-sulfone ADC discovery spurred the development of a wide range of rebridging systems, with the apex goal of creating ADCs with a DAR of 4. For example, dihaloacetones **7** have been utilized for rebridging by alkylation, whereby the ketone-containing rebridged antibody product can be reacted with hydroxylamine-containing payloads to afford oxime-linked conjugates (Scheme 3c) [29–30]. Additionally, dibromomaleimides **8** [31–32] and dibromopyridazinediones **9** (Scheme 3d) [33] bearing payloads at the *N*-position can rebridge cysteines through an addition–elimination mechanism. These reagents have been extensively explored for protein conjugates, especially as ADCs [34]. Similarly, divinylpyrimidines **10** [35] and arylenedipropiolonitriles **11** [36] have also been investigated for analogous purposes but via a conjugate addition mechanism (Scheme 3e). Exploiting the unique oxidation state of phosphorous, diethynylphosphonites **12** also utilize conjugate addition to provide a versatile three-point conjugation platform [37].

**Scheme 4:**
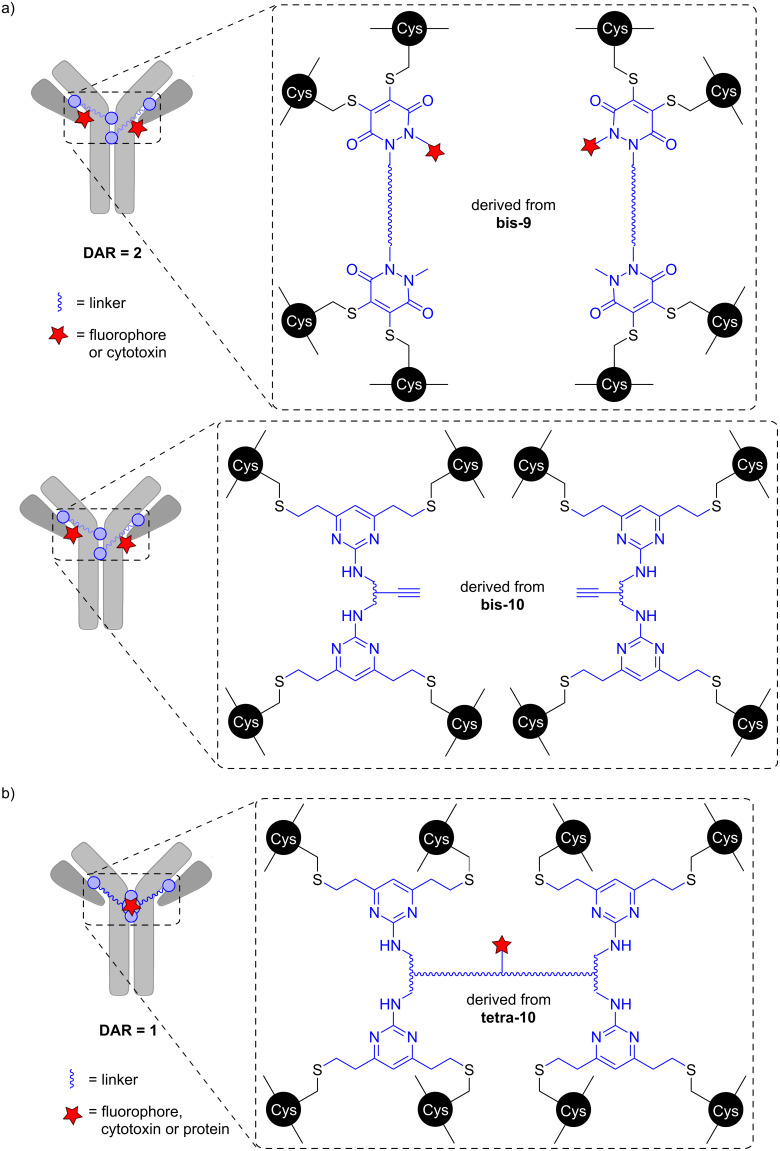
Multi-disulfide rebridging technologies.

Related technologies have been adapted to link multiple disulfide residues simultaneously. Using bis-dibromopyridazinediones **bis-9** [38] or bis/tetra-divinylpyrimidines **bis/tetra-10** [39], the toolbox of native sequence modification has been expanded to enable the construction of homogeneous conjugates bearing 1 or 2 payloads (Scheme 4). In the case of tetra-divinylpyrimidines **tetra-10**, antibody conjugates have been constructed that bear a single fluorophore, cytotoxin or SpyCatcher protein [40]. Despite the considerable modification of the chemical space around the antibody’s hinge region, antigen affinity and antibody stability are largely retained.

**Scheme 5:**
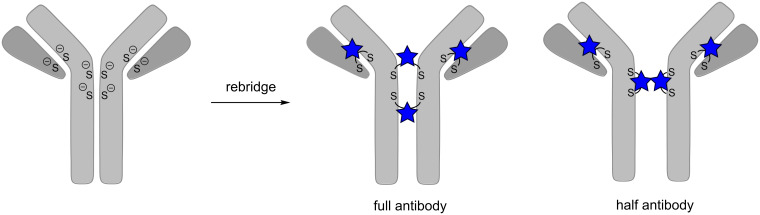
Formation of full- and half-antibody as a result of disulfide rebridging.

Translating disulfide chemistries into therapeutic applications requires optimisation of key parameters including reaction kinetics, minimisation of reaction steps, conjugate stability and solubility. Furthermore, disulfide rebridging carries an inherent risk of scrambling, compromising homogeneity. In the case of DAR 4 ADCs, this approach enables conjugation with fairly homogeneous loading, yet heterogeneity within populations of the same DAR species exists, with bis-reactive reagents forming a mixture of half- and full-antibody conjugates through intra- and interchain rebridging, respectively (Scheme 5). Nevertheless, the two halves of the half-antibody are still held tightly together by non-covalent interchain interactions. It remains unclear whether half-antibody structures are less stable or efficacious than their full counterparts [41], however, it can be posited that the technology has not yet entirely fulfilled its original promise of completely homogeneous conjugate generation.

More broadly, this strategy is limited by its reliance on native cystine residues, making it unsuitable for proteins that lack disulfide bonds or that aggregate upon reduction. Its application to endogenous protein labelling is also restricted, as disulfide reduction may destabilise native protein structures, limiting uses in biological research. Perhaps, to expand applicability, future efforts may target alternative functional pairs, such as aromatic–π systems and the *N-*/*C-*termini (which are often in close proximity). One related example is cross-linking histidine residues by use of monosulfone [42]. For in-depth discussions of rebridging technologies, see references [34,43–45].

#### Affinity-guided protein modifications

Affinity-guided protein labelling, also known as affinity-directed conjugation, is a native-sequence modification strategy founded on supramolecular recognition. It employs a bifunctional probe composed of (1) an affinity ligand that binds to a localised region of a target protein, and (2) a reactive moiety, either a pre-installed electrophile or a catalytic unit that generates one. Once bound, the reactive group is transferred to the target protein, enabling covalent modification. This concept has become a cornerstone of biological research and therapeutic development, with applications including in vitro systems, live cells and animal models.

**Scheme 6:**
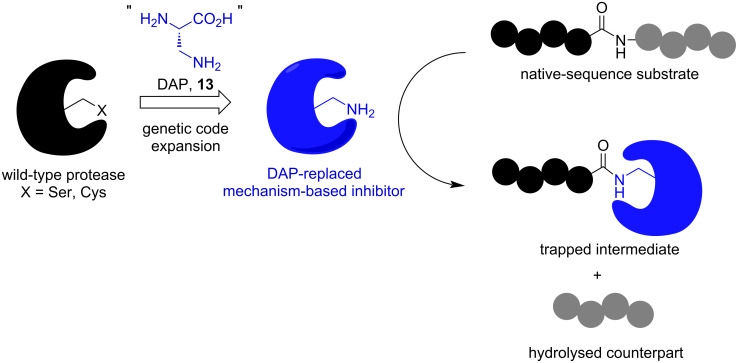
Use of 2,3-diaminopropionic acid (DAP, **13**) for native-sequence substrate profiling.

The earliest form of affinity-guided labelling traces back to mechanism-based inhibitors, which exploit heightened reactivity of active-site residues for enzyme modification [46]. This concept evolved into activity-based protein profiling (ABPP), pioneered by the Cravatt group, where peptides or small molecules bearing electrophilic groups covalently react with active-site residues, enabling phenotype-driven functional profiling in a complex biological environment [46]. In a related concept, the Chin group reported the genetic incorporation of the unnatural amino acid 2,3-diaminopropionic acid (DAP, **13**), a nucleophilic amine that has a relatively low p*K_a_* (6–8) and is structurally analogous to serine or cysteine, enabling identification of native-sequence protease substrates through site-specific hydrolysis and isopeptide-bond formation (Scheme 6) [47].

Labelling residues on protein surfaces is noticeably more challenging due to a lack of residues with heightened reactivity. Substantial progress has been made to address this issue, with current strategies broadly classified into non-catalytic and catalytic approaches.

**Scheme 7:**
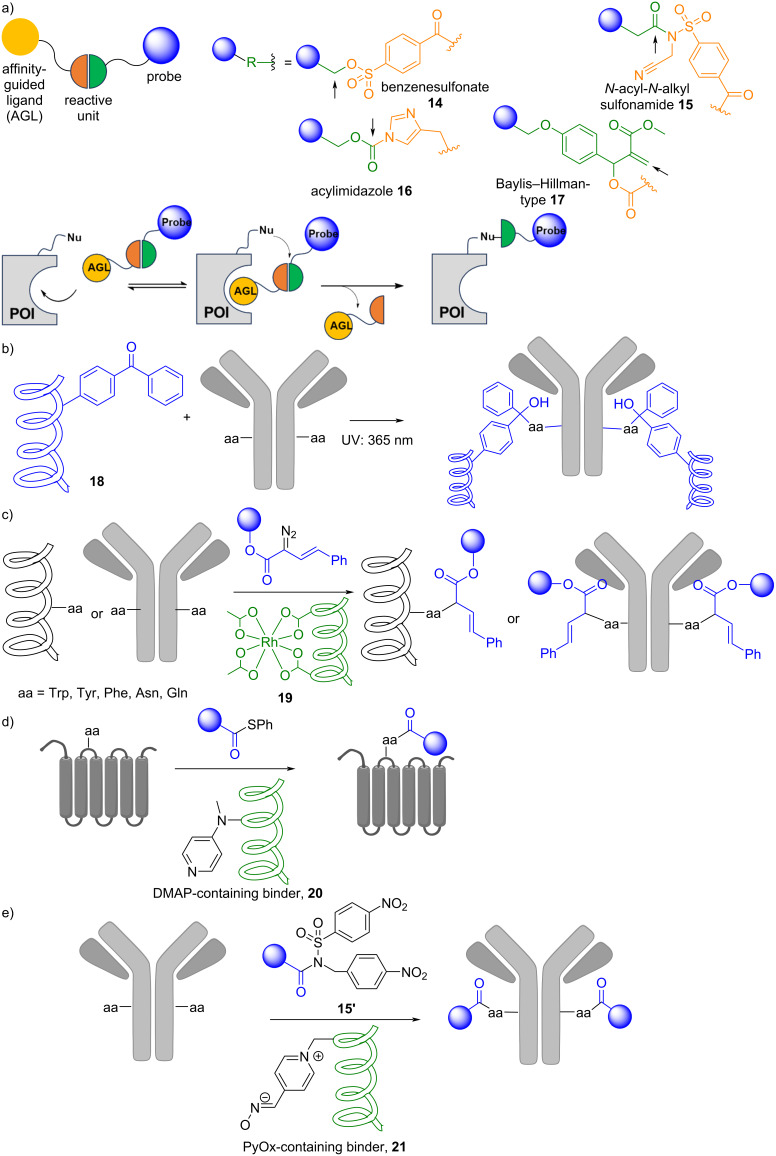
Affinity-guided labelling. a) Reagents and catalysts appended to the binder include electrophilic motifs **14**–**17** as well as b) benzophenone **18**, c) rhodium complexes **19**, d) 4-dimethylaminopyridine **20** (DMAP), and e) pyridinium aldoxime **21** (PyOx).

Non-catalytic approaches often involve tethering a reactive electrophile to a protein-binding ligand, thereby positioning the electrophile near a neighbouring nucleophilic residue. Example electrophiles include 4-methylbenzenesulfonates **14**, *N-*acyl-*N-*alkyl sulfonamides **15**, acylimidazoles **16**, Baylis–Hillman-type **17** reagents, among others (see reviews [48–49] for a comprehensive summary; Scheme 7a). Highlighting the challenges in pursuing both reactivity and stability, the *N*-acyl-*N-*alkyl sulfonamide possesses superior kinetics [50], whereas tosylation is one of the few procedures compatible with red blood cells and animal labelling [51]. Alternatively, benzophenone **18**, which can be incorporated in the form of an unnatural amino acid, can form a UV-induced diradical for protein modifications (Scheme 7b) [52–55]. Proteins, including IgG (trastuzumab) [56], EGFR [57], the adenosine A1 receptor [58] and P2X7 [59], have been labelled in vitro and/or in cellular systems.

Catalytic approaches generate reactive species in situ, thus enhancing both selectivity and efficiency. A notable example involves rhodium (Rh)-based catalysis, which mediates carbene transfer to modify electron-rich residues (Scheme 7c). This was first demonstrated in a coil dimer (Jun/c-Fos), where a dirhodium complex on one subunit enabled selective labelling of tryptophan, tyrosine and phenylalanine residues on the partner subunit [60]. This Rh-based method (**19**) was later adapted for modification of an asparagine (Asn) residue in the Fc domain of trastuzumab [61]. Rhodium has also been used in visible-light-triggered single-electron transfer (SET) reactions, enabling labelling under mild conditions with spatiotemporal control. Another strategy employs a 4-dimethylaminopyridine-containing binder **20**, in which dimethylaminopyridine (DMAP) acts as nucleophilic base catalyst mediating acyl cation transfer, that was successfully applied to label lectins and cell-surface receptors (HER2 and EGFR; Scheme 7d) [62–63]. More recently, genetic code expansion has enabled pyridinium aldoxime **21** (PyOx) incorporation into protein scaffolds (Scheme 7e) [64]. Acting as a catalytic centre for acyl cation transfer, PyOx reacts with a selected *N-*acyl-*N-*alkyl sulfonamide **15’** to generate a reactive ester intermediate. This strategy has successfully modified lysine residues in model proteins Z-DM, Fc domains and trastuzumab.

Affinity-guided protein labelling has proven highly valuable for both biological and translational research. Approaches involving genetic code expansion are especially promising, as this technique is now widely implemented in most molecular biology laboratories. The diversity of binder templates has also expanded rapidly with advances in AI-based and high-throughput technologies [65–77]. Nevertheless, design considerations remain critical; for example, benzophenone could cross-link an entire binder to its target protein [52–55], whereas incorporation of DAP requires photodeprotection [47]. Furthermore, all current proximity-directed reagents, including catalytic systems such as the reactions mediated by DMAP and PyOx (Scheme 7d and 7e), still require extensive screening and substantial stoichiometric quantities, which significantly limits their up-scaling potential. For further discussion, including non-specific labelling for protein interactome analyses, see reviews [48–49].

#### Small molecule residue modifications

**Scheme 8:**
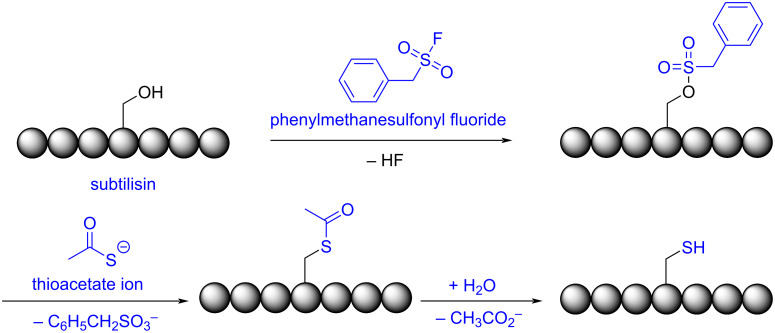
Chemical sequence to convert subtilisin serine residue into a cysteine analogue.

Small-molecule reagents are generally user friendly and scalable, with many already commercially available. Their small size makes them atom-economical but achieving site-specificity remains challenging since identical residues are often distributed across protein surfaces. The first example was Sanger’s reagent (1-fluoro-2,4-dinitrobenzene, **1**) mentioned above [6]. Other early examples include cysteine modification of ribonuclease A (RNase A) using iodoacetic acid [78]. Early enzyme engineering also explored single-atom modifications, converting subtilisin’s serine residue into a cysteine analogue through a three-step chemical sequence (sulfonylation → thioacetate substitution → hydrolysis; Scheme 8) [79], laying the groundwork for ligase development [80–81]. Lysine residues are abundant and typically solvent-exposed [82], hyper-reactive variants have been reported, and have led to the development of tools such as “K-lock” [83–85]. Unfortunately, the reactivity difference is often not sufficient for site-specific modifications. Consequently, recent effort has shifted towards targeting alternative less-frequent amino acid residues [82], as well as novel mechanisms such as linchpin-directed modifications summarized below.

**Scheme 9:**
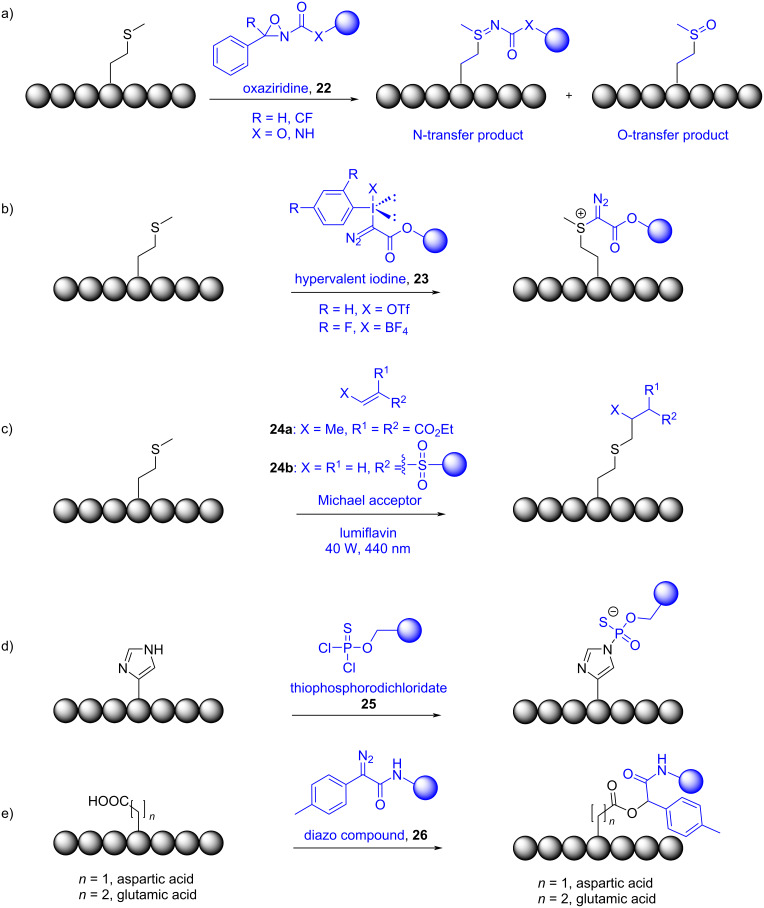
Examples of small molecule reagents. Modifications of a–c) methionine by oxaziridine **22**, hypervalent iodine **23**, Michael acceptors (**24a/b**), d) histidine by thiophosphorodichoridate **25,** e) aspartic and glutamic acid by diazo-containing compounds **26**.

**Scheme 10:**
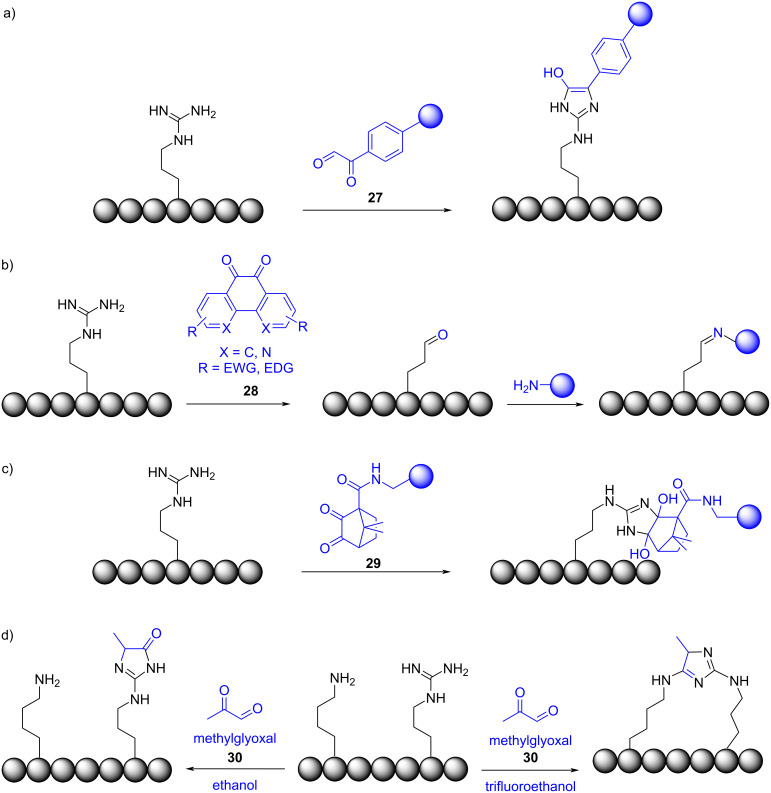
Labelling of arginine by reagents carrying a) glyoxal **27**, b) phenanthrenequinones **28**, c) DKPA **29** groups and d) methylglyoxal (**30**).

Various chemoselective methods for alternative residues have recently emerged. Methionine has gained traction as a labelling site due to its electron-rich thioether sulfur atom, which possesses versatile bond-forming capacity. The redox-activated chemical tagging (ReACT) platform employs oxaziridines **22** to form stable sulfimide (S=N) linkages, with applications in ADC development, radiolabelling and ROS/Ca^2+^ imaging (Scheme 9a) [86–91]. Methionine can also be modified via sulfonium conjugates using hypervalent iodine (**23**, Scheme 9b) [92] or photoredox approaches (**24a**/**b**, Scheme 9c) [93]. Histidine has been targeted using thiophosphorodichloridate reagents **25**, as shown with hexahistidine-tagged proteins (Scheme 9d) [94]. Aspartate and glutamate, while weakly nucleophilic, can undergo *O*-alkylation via diazo compounds **26** (Scheme 9e) [95–96]. Arginine, traditionally difficult to modify due to its high p*K*_a_ values, can be targeted with dielectrophilic glyoxal-based amidine formation (**27**, Scheme 10a) [97–100], aldehyde generation via phenanthrenequinones **28** (Scheme 10b) [101], diketopinic acid (DKPA, **29**) conjugation (Scheme 10c) [102], and methylglyoxal (**30**)-mediated arginine–lysine cyclisation (Scheme 10d) [103].

**Scheme 11:**
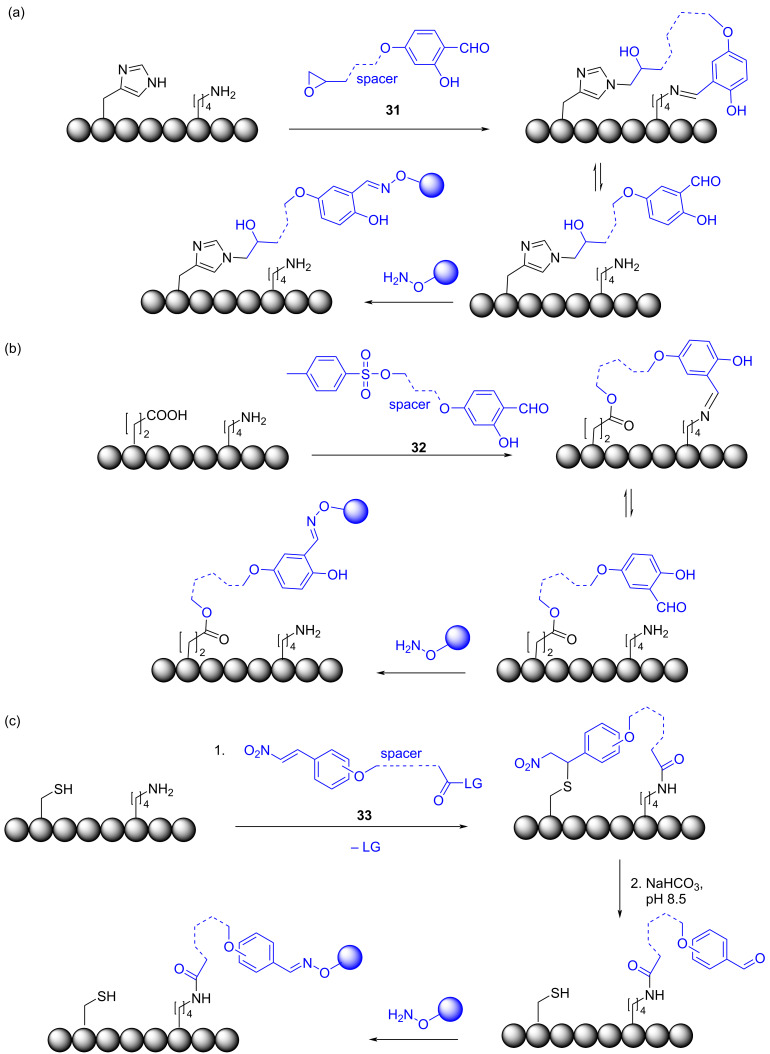
Examples of linchpin-directed modification targeting a) histidine, b) aspartic acid, and c) lysine residues via transient interaction with neighbouring residues, including lysine-reacting *o*-hydroxybenzaldehyde reagents **31**–**32** and cysteine-reacting β-nitroalkene reagent **33**.

A different approach has been the development of linchpin-directed modification (LDM) [104]. These bifunctional reagents contain two reactive groups separated by a spacer: one engages in a reversible interaction with a common surface residue, while the other is an irreversible chemical handle that covalently modifies a nearby target residue such as histidine (Scheme 11a) [105], aspartate (Scheme 11b) [106] and lysine (Scheme 11c) [107]. For example, reagents containing *o*-hydroxybenzaldehyde (**31**, **32**) and β-nitroalkene (**33**) can reversibly engage lysine and cysteine residues, respectively, while a proximal electrophile, such as an epoxide, sulfonate ester or ester, enables targeted covalent modification of amino acids [105–107]. In addition to the choice of electrophiles, selectivity can be refined by tuning the length and flexibility of the linker, which controls the spatial proximity of the reactive groups. While simple, the LDM approach, with appropriate use of linker, could label exclusively cytochrome C out of a mixture of proteins (ubiquitin, RNaseA, myoglobin, ubiquitin, lactalbumin, lysozyme C, α-chymotrypsinogen A). This approach can be extended to native-sequence labelling of Trastuzumab, enabling site-specific histidine modification of the Fc region, albeit at a modest yield (37%). Another related and noteworthy technology is the cysteine-to-lysine transfer reaction, which operates via a mechanism analogous to native chemical ligation in Fab fragments [108].

Together the advances of small-molecule modifications provide powerful strategies for site-selective modification of otherwise intractable residues, as summarised in a comprehensive review [109]. However, most new conjugation reagents are not yet commercially available, requiring in-house synthesis, and the resulting conjugates often exhibit uncertain and incompletely characterised stability. Moreover, depending on the conjugated epitope, modifications can cause loss of function or aggregation, underscoring the need for further innovation.

#### Post-translational modification (PTM) chemical labelling

**Scheme 12:**
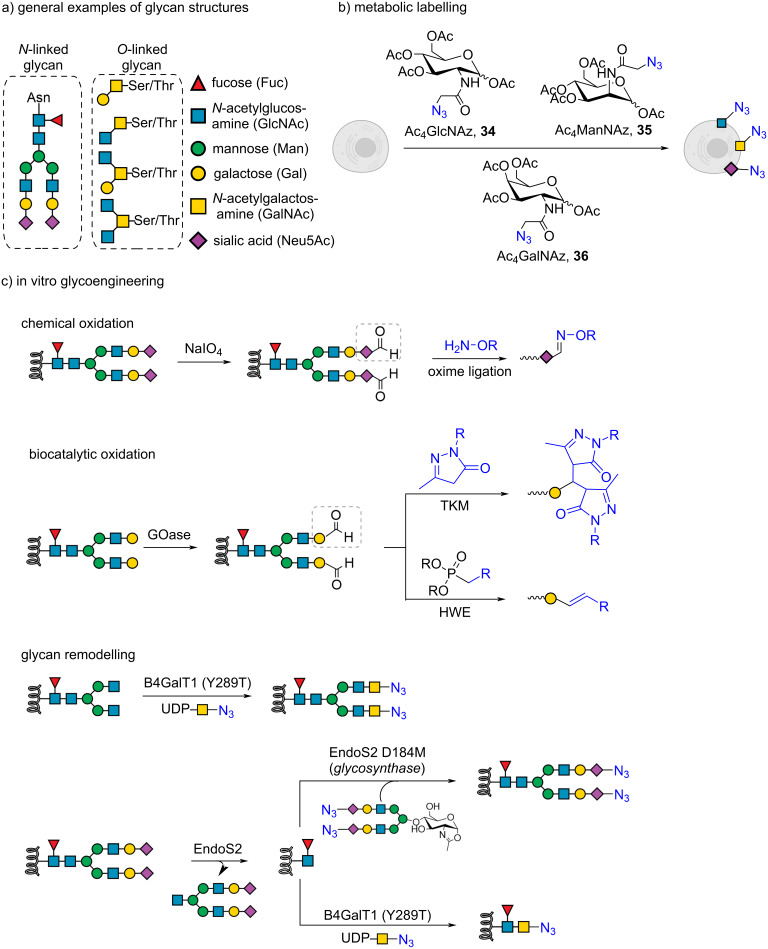
Overview of glycan-directed strategies for site-specific protein modification. a) Structural motifs of *N-*linked and *O*-linked glycans commonly targeted in bioconjugation; b) metabolic glycoengineering introduces azido-functionalised sugars such as Ac_4_GlcNAz (**34**), Ac_4_ManNAz (**35**) and Ac_4_GalNAz (**36**) into cellular glycosylation pathways for bioorthogonal labelling; c) in vitro glycoengineering employs chemical (e.g., periodate) and/or enzymatic remodelling including the use of engineered oxidases (galactose oxidase), glycosyltransferases (β4GalT1 Y289L) or glycosynthases to install reactive handles on native glycans, enabling precise conjugation for applications such as antibody–drug conjugates (ADCs).

Post-translational modifications (PTMs) refer to chemical changes to a protein in-cell after it is synthesised by the ribosome, encompassing over 650 types of transformations, including phosphorylation, methylation, acylation, ubiquitination and glycosylation [110]. While numerous naturally occurring PTMs have been repurposed for bioconjugation chemistry [111–113], manipulating the composition of native protein glycans, known as glycoengineering, has become a privileged strategy for site-specific modification without altering the protein’s native amino-acid sequence. This approach requires the target protein to be *N*- or *O*-glycosylated (Scheme 12a). *N*-Linked glycans, prominently present on antibodies, have been extensively exploited for antibody–drug conjugate (ADC) development, while *O*-linked glycans remain less explored but represent an emerging opportunity.

Glycan remodelling strategies are typically dissected into two main branches: metabolic and in vitro glycoengineering. Metabolic glycoengineering introduces a non-natural sugar precursor, such as Ac_4_GlcNAz (**34**), Ac_4_ManNAz (**35**) and Ac_4_GalNAz (**36**, Scheme 12b) into living cells. These analogues enter the cellular glycosylation pathway, thereby incorporating azide motifs into native glycans for chemical attachment through bioorthogonal chemistry (e.g. strain-promoted azide–alkyne cycloaddition/SPAAC) [114–119]. While this strategy enables visualisation and derivatisation of native glycoproteins in their natural environment, metabolic labelling rarely achieves complete functionalisation due to competition from natural substrates, limiting its utility for recombinant protein production. GALaXy technology is a notable exception [120–121]; however, as the technology requires a tag sequence, it is outside the scope of this review.

In vitro glycoengineering, a technology that uses engineered glycoenzymes such as glycosyltransferases and glycosidases to modify glycans, has been widely adopted for native protein labelling, with particular emphasis on antibodies (Scheme 12c). This section aims to give an overview of general strategies adopted for in vitro glycoengineering, and the reader is directed to specialised reviews for in-depth discussions [122–124]. Periodate oxidation of the non-reducing end of *N*-glycans is perhaps the oldest and most straightforward approach to generate an aldehyde biorthogonal handle on a glycoprotein, which can then be subjected to conjugation such as hydrazone or oxime ligation [125–128]. However, while this approach is still a valid strategy for protein functionalisation, potential undesired side reactions such as amino acid oxidation need to be carefully taken into consideration. Use of galactose oxidase (GOase) to site-specifically oxidise galactose to galactoaldehyde on the non-reducing end of a glycan represents a mild alternative, as showcased by its combination with Horner–Wadsworth–Emmons (HWE) chemistry in a one-pot oxidation olefination strategy [19], as well as its implementation with tandem Knoevenagel–Michael addition (TKM) conjugation to enable production of glycoengineered high DAR ADCs [129].

Qasba’s engineered bovine galactosyltransferase (β4GalT1(Y289L)) is an elegant example of using glycosyltransferases to remodel glycans of target protein with non-natural sugars (e.g., GalNAz), leading to its wide adoption in academic glycan remodelling research and the development of commercial kits [130–132]. Based on structural data, Tyr289 of bovine β4GalT1 was found to be important for determining UDP-Gal specificity and its mutation to a less hindered leucine residue broadens the scope of azido-carrying nucleotide sugar analogues (UDP-GalNAz), thereby introducing the bioorthogonal handle on the glycan [131–132]. Similarly, sialyltransferases, featuring remarkable substrate promiscuity, have also been exploited to install non-natural sugars on glycans [115–119].

Glycosynthases are engineered variants of glycosidases widely used for glycan remodelling. By substituting the catalytic nucleophile with a non-nucleophilic residue, the engineered enzyme can catalyse glycosidic bond formation with abolished hydrolase activity [133–137]. One such example is the mutation of endoglycosidase S2 (EndoS2) catalytic Asp184 residue into Met (EndoS2 D184M), generating a variant capable of transferring a pre-assembled azido-glycan oxazoline donor substrate onto an antibody with high homogeneity [137]. However, while the elegance of this strategy is undeniable, synthesis of glycan oxazolines remains challenging thus limiting its wider adoption. In an alternative strategy, wild-type EndoS2 can be used for antibody glycan trimming, before the aforementioned β4GalT1(Y289L) can be used to mediate GalNAz transfer generating homogenised *N-*glycan, a patented technology called (GlycoConnect^®^) used in ADC manufacturing [138–139].

In summary, glycan-directed labelling excels by omitting engineering or denaturation of the protein substrates. Often, the protein function is mostly preserved, for example, the antigen-binding (Fab) region of antibodies is free of glycan modification. The chemical or enzymatic steps are generally mild (room temperature, aqueous buffer) yet highly specific. However, the main limitation is that only glycosylated proteins (especially IgGs) can be targeted. Intrinsic glycan heterogeneity can also lead to incomplete functionalisation. Biological activity of the modified protein, recyclability and manufacturing cost of the enzymes also need to be carefully considered, as costs can easily balloon in process development. Nonetheless, the use of glycan remodelling is expanding for applications such as site-specific protein labelling, with ADC development exemplifying its significant potential.

#### Enzyme-mediated modifications

**Scheme 13:**
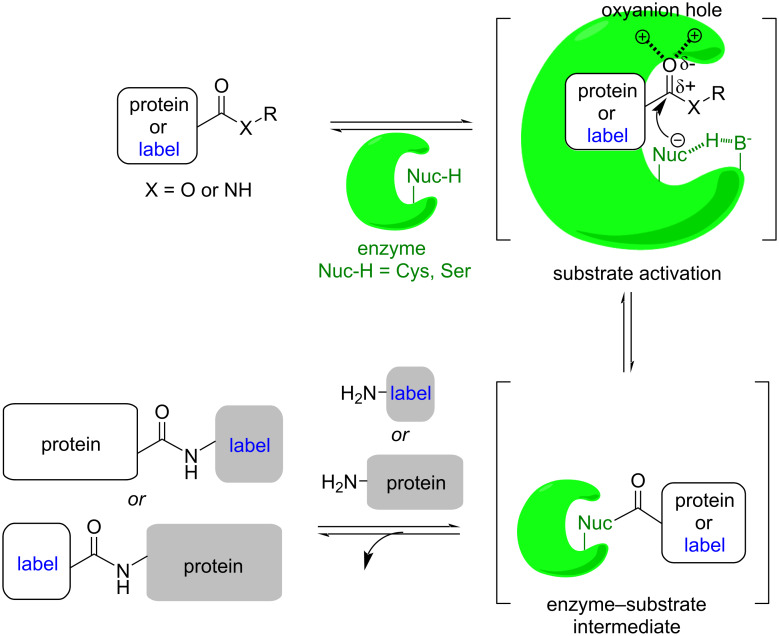
Generic reaction mechanisms recruited by various native-sequence modifying enzymes.

Enzymes are highly attractive tools for native-sequence protein modification, offering exquisite spatial precision under mild, biocompatible conditions and easy access via recombinant technologies. Their catalytic activity supports scalability, making them suitable for both academic and industrial applications. Moreover, enzymes enable a broad spectrum of chemistries, targeting both amino acid sidechains and the protein backbone [140–142]. Despite these advantages, the repertoire of enzymes applied for native-sequence modifications remains limited. The following examples illustrate recent advances in recruiting enzymes for native-sequence labelling, all of which rely on reaction mechanisms that activate carbonyl groups (Scheme 13).

**Scheme 14:**
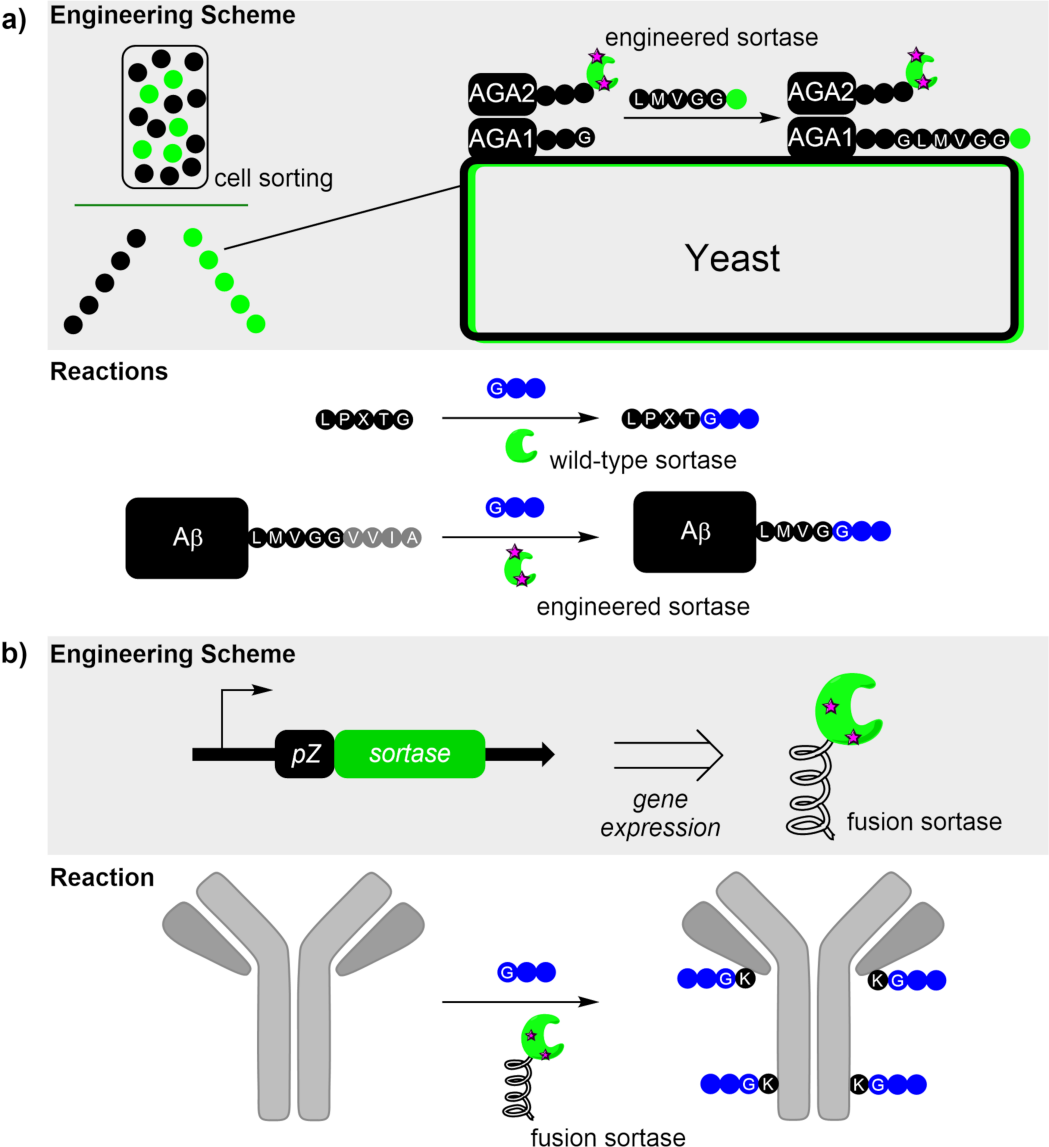
a,b) Engineered biocatalysts including sortase. Proteins AGA1 and AGA2 are surface proteins naturally found on yeast surface.

One of the most studied protein-modifying enzymes is sortase A. Recognising the LPXTG motif, the enzyme cleaves the Thr–Gly peptide bond to form an acyl intermediate that is ligated to an incoming primary amine nucleophile, such as an *N-*terminal glycine or lysine ε-amine (Scheme 14a) [143]. Native proteins with an *N-*terminal glycine and sufficient flexibility can thus be directly modified by sortase. Optimisations using depsipeptides or coupling strategies have further improved conversion efficiency and reduced reagent consumption [144]. Due to its ease of preparation, large libraries of sortase variants have been generated and screened to expand substrate scope [143]. In one example, sortase variants and *N-*terminal polyglycine tags were fused to yeast surface proteins AGA-1 and -2, respectively, while peptides carrying GG alternative motifs and reporter tags were introduced as substrates. Active variants covalently attached the reporter to the yeast surface, allowing isolation via FACS (Scheme 14a). After 16 rounds of selection from a library of 10^7^ variants, enzymes capable of recognising LMVGG, a sequence absent from wild-type specificity but present in endogenous amyloid protein Aβ in human cerebrospinal fluid, were obtained [145].

An affinity-guided strategy was used to evolve sortase A variants for antibody conjugation [146]. Exploiting the ability of sortase to accept the ε-amino group of lysine, the enzyme was fused to an antibody Fc-binding domain protein (protein Z/pZ), positioning it near surface-exposed lysine on the target antibody for isopeptide bond modification (Scheme 14b). These fusion constructs, developed based on the concept of affinity-guided labelling, were immediately active and served as starting points for further engineering. After only one to three rounds of mutagenesis via colony screening with glycosylated trastuzumab and cetuximab, variants 1M (N127K) and 3M (T156S/D170E/D176E) with enhanced labelling activity were isolated. Although this system offers switchable activity by removing calcium ions from the buffer, it achieves only moderate site-specificity; labelling was found in K5, K123, K135, K292, and K441 on the heavy chain and K207 on the light chain of cetuximab, out of ≈80 lysine residues. However, antigen binding was largely unaffected, and a range of payloads including TAMRA, biotin and the cytotoxic drug MMAE could be incorporated to yield a DAR ratio of 2–3 [146].

**Scheme 15:**
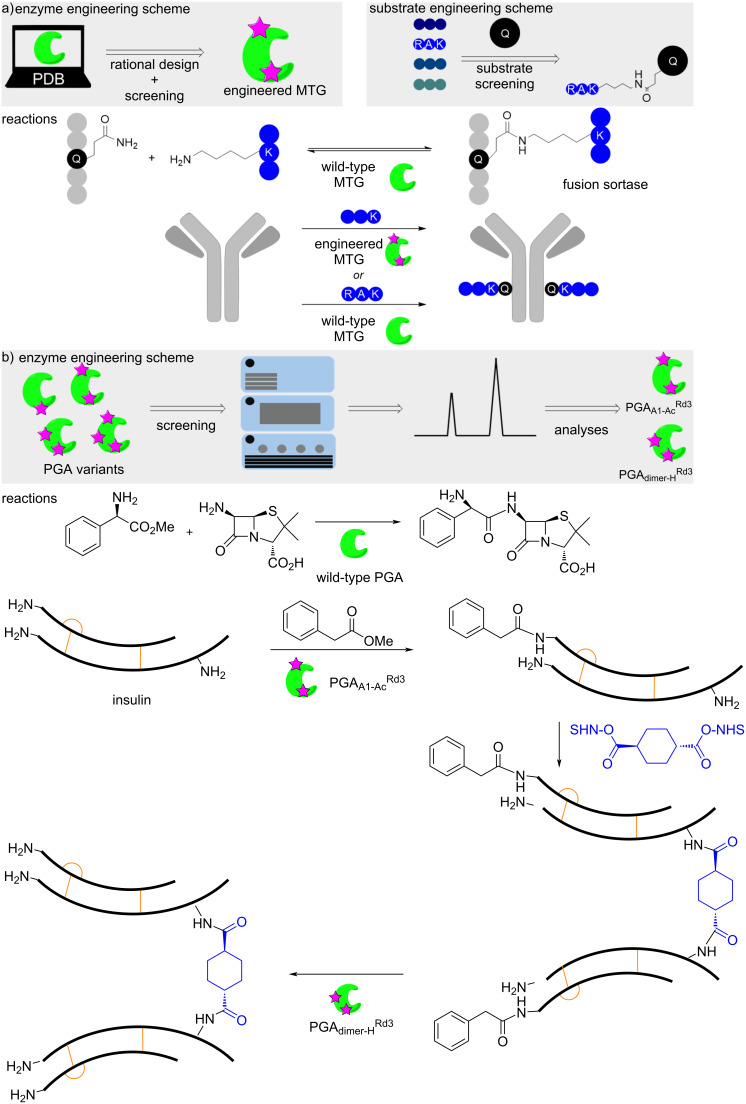
Engineered biocatalysts include a) microbial transglutaminase (MTG) and b) penicillin G acylase (PGA).

Microbial transglutaminase (MTG), which forms isopeptide bonds between a target protein and glutamine (or other small primary amides) is another frequently used enzyme for bioconjugation. The primary amide in glutamine is converted into an acyl intermediate that is subsequently attacked by amine nucleophiles such as lysine ε-amines and glycine α-amines. Although MTG can modify Gln295 on the antibody Fc, this residue is close to the Asn297 glycan and hence normally requires deglycosylation or N297A mutation for enzyme access, both of which reduce structural stability and diminish antibody-dependent cell-mediated cytotoxicity (ADCC) [147–148]. To enable labelling of fully native antibodies, two complementary strategies have been developed (Scheme 15a). First, rational engineering of MTG yielded active-site mutants, with modifications located in a loop projecting into the catalytic pocket (G250S), which enhanced reactivity towards native antibody Asn297 [149]. Further optimisation delivered double variants G250S/E300D (“S2”) and G250S/K327T (“S13”), both capable of generating ADCs with DAR close to 2.0. Second, substrate engineering revealed that MTG preferentially processes positively charged peptides, with an RAK-containing motif supporting efficient ligation to fully glycosylated antibodies [150]. Collectively, these enzyme- or substrate-engineering approaches enabled native-state labelling of multiple antibodies (avelumab, matuzumab, cetuximab, and rituximab), producing stable conjugates with favourable biodistribution [149–150].

Another example is the engineering of the hydrolase penicillin G acylase (PGA) for the selective modification of insulin [151]. After screening 100 commercially available PGA homologs and initial optimisation for expression and handling, *Kluyvera cryocrescens* PGA was chosen for directed evolution (Scheme 15b). The goal was to selectively protect or deprotect key amines on insulin, including the *N-*terminal glycine of chain A, the *N-*terminal phenylalanine of chain B and the ε-amino group of lysine B29. Using a colony picker and UPLC analysis, mutations were introduced within the binding pocket (to enhance localised recognition of insulin) and at distal sites (to improve stability and solvent tolerance). The engineered enzyme PGA_A1-Ac_^Rd3^ selectively introduced a benzoyl group at the *N-*terminus of chain A, whereas PGA_dimer-H_^Rd3^ catalysed deprotection enabling formation of dimerised insulin candidates under mild chemoenzymatic conditions. Chromatography-free precipitation gave yields up to >90%, and scalability was demonstrated at >120 grams.

These enzyme-based examples are elegant but not without limitations, largely due to the labour-intensive workflows and the intrinsic design constraints associated with the use of naturally derived enzymes. The sortase variant engineered to recognise amyloid proteins was highly selective for the altered sequence (*K*_M_ = 128 µM) but achieved only modest catalytic efficiency (143 M^−1^ s^−1^, compared with 10^3^–10^4^ M^−1^ s^−1^ in wild-type), likely because the FACS-based screening was based on single-turnover events. The sortase A-protein Z system has limited site specificity; it also requires equimolar enzyme-to-antibody ratios and additional purification steps. MTG, similarly, requires near-stoichiometric enzyme to function, restricting scalability; this enzyme is also produced as a zymogen and requires activation post-purification, complicating the engineering workflow. PGA optimisation benefited from the simplicity of insulin, which contains only three primary amines whose labelling can be readily resolved by UPLC, but such strategies become impractical for larger, lysine-rich proteins. For a broader overview, Zhu et al. [141] provide a comprehensive summary of recent advances in engineering amide-bond-forming enzymes for modifications, including asparaginyl endopeptidase (AEP), omniligase and other emerging biocatalysts.

## Comparative Analysis, Outlook and Future Directions

In Table 1, we compile a bird’s-eye overview of current site-specific native-sequence protein modification strategies. The check marks represent best-case scenarios reported in the literature. Native chemical ligation offers high precision for *N*-terminal cysteine modifications and synthesis of diverse proteins, whereas disulfide-rebridging approaches provide scalability due to the relative ease of reagent preparation (see above for detailed discussion). Overall, however, the success of any given method is often case-dependent, reflecting the diversity of protein and peptide sequences and structures. Factors such as protein preparation can also influence labelling efficiency; this is often the case for *N*-terminal and glycan modifications where composition of the protein may vary with the recombinant host. Protein precipitation and unintended side reactions, which are often underreported, can further complicate the anticipated outcome. The limitations listed in Table 1 reflect intrinsic constraints of the tool design, such as the requirement for accessible cysteine or disulfide motifs in the corresponding technology. For modifications by naturally derived enzymes, a balance often needs to be struck between substrate promiscuity and selectivity. In general, highly substrate-promiscuous enzymes require more complex preparation procedures, which can hinder their engineering, whereas highly selective enzymes are easier to handle but depend on defined recognition sequences. Ultimately, we found that there is no single strategy simultaneously fulfilling all criteria, and trade-off(s) are inevitable. Hence, we hope this overview will help readers identify opportunities for developing new strategies that address these current limitations.

**Table 1:** Comparison of existing site-specific, native-sequence protein modification strategies.

	Cysteine modification	*N*-Terminal labelling	Affinity-guided chemistry	Disulfide- rebridging technology	Small molecule	Glycan modification	Existing enzymes

precision	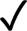	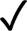	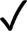	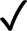	limited positional control, though often residue specific	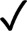	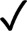
scalability	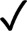	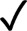	complex reagents used in stoichiometric portion	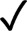	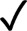	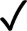	may require stoichiometric portion
usability	only for low-abundance cysteine	highly sequence dependent	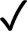	only for low abundance disulfide motif	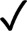	glycan must be present	may require recognition motif may require extensive engineering

**Scheme 16:**
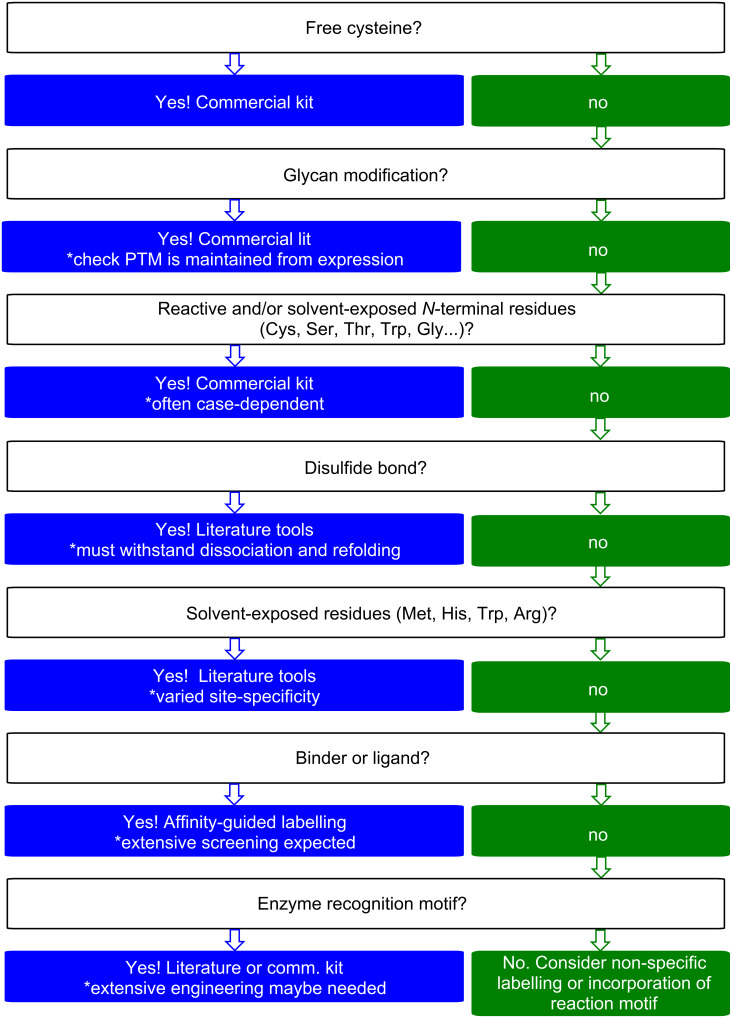
Selection scheme of a suitable native-sequence labelling tool.

For readers seeking to adapt existing technologies for protein modifications, we present a generic decision framework for selecting a site-specific native-sequence protein modification strategy (Scheme 16). The first step is to evaluate cysteine content, as these residues are the most established targets with a wide range of stable, commercially available reagents. Similarly, if the target protein is glycosylated, numerous glycoengineering strategies and commercial tools can be considered. In the absence of free cysteines or glycans, the *N-*terminus can be chemically targeted, particularly when it contains dinucleophilic residues such as cysteine, serine, threonine, or tryptophan. Enzymes such as sortase (for glycine-containing motifs), omniligase and AEP can be also considered as tools when the *N*-terminal modification site is sufficiently solvent exposed. Alternatively, disulfide bonds may provide modification sites through rebridging, provided that the protein tolerates reduction-and-rebridging reaction sequences. If these options are unsuitable, other solvent-exposed nucleophilic residues (e.g., methionine, histidine, tryptophan, tyrosine or arginine) can be targeted, although such reagents are often prepared in-house and may lack positional control. When high-affinity ligands or binders are available, affinity-guided conjugation strategies can be implemented, though probe design and optimisation are often required. Finally, if an enzyme recognition motif is available, biocatalytic approaches can be pursued, particularly for commercially accessible enzymes, although at times they require in-house screening and/or engineering to achieve the desired specificity. As a general guideline, the final choice depends on multiple factors, including protein properties (sequence, flexibility, post-translational modification status, stability) and reagents availability (commercial reagents, in-house preparations, or enzyme accessibility).

In summary, a fundamental barrier associated with native-sequence protein modifications arises from the presence of multiple identical residues within a protein, each complicated by its specific structural context. Moreover, all chemistries must operate under biocompatible conditions, restricting the scope of viable options. Nevertheless, continued innovations at the interface of synthetic chemistry and biology are expected to address these barriers, enabling the development of broadly adaptable platforms for the efficient production of modified proteins.

## Data Availability

Data sharing is not applicable as no new data was generated or analyzed in this study.
